# Guidelines for the description of rhizobial symbiovars

**DOI:** 10.1099/ijsem.0.006373

**Published:** 2024-05-14

**Authors:** Esperanza Martinez-Romero, Alvaro Peix, Mariangela Hungria, Seyed Abdollah Mousavi, Julio Martinez-Romero, Peter Young

**Affiliations:** 1 Center for Genomic Sciences, UNAM Cuernavaca, Morelos, Mexico; 2 Instituto de Recursos Naturales y Agrobiología, IRNASA-CSIC, Salamanca, Spain; 3 Interacción Planta-Microorganismo, Universidad de Salamanca, Unidad Asociada al CSIC por el IRNASA, Salamanca, Spain; 4 Embrapa Soja, Soil Biotechnology Laboratory, Londrina, Paraná, Brazil; 5 Department of Biology, University of Turku, 20014 Turku, Finland; 6 Department of Biology, University of York, York YO10 5DD, UK

**Keywords:** host specificity, nitrogen fixation, nodC, nodulation, symbiosis, symbiosis islands, symbiosis plasmids, symbiovar

## Abstract

Rhizobia are bacteria that form nitrogen-fixing nodules in legume plants. The sets of genes responsible for both nodulation and nitrogen fixation are carried in plasmids or genomic islands that are often mobile. Different strains within a species sometimes have different host specificities, while very similar symbiosis genes may be found in strains of different species. These specificity variants are known as symbiovars, and many of them have been given names, but there are no established guidelines for defining or naming them. Here, we discuss the requirements for guidelines to describe symbiovars, propose a set of guidelines, provide a list of all symbiovars for which descriptions have been published so far, and offer a mechanism to maintain a list in the future.

## Introduction to symbiovars

The outstanding characteristic of rhizobia is their capacity to form nitrogen-fixing nodules in legume plants. The genes involved in this process have been studied for a long time, especially the *nod* genes involved in nodulation [1]. These *nod* genes, as well as *nif* and *fix* genes (involved in nitrogen fixation), are found in symbiosis plasmids or islands, together with other symbiosis genes and genes that encode diverse secretion systems [2,3]. Notably, symbiosis genes constitute a small fraction of all genes found in symbiosis plasmids or islands, which contain other genes expressed in free-living conditions [4] or for environmental adaptability [3,5].

Symbiosis genes are part of the accessory and mobile bacterial genome that may be gained by horizontal gene transfer (HGT) or lost [6,8]. Indeed, there are many non-symbiotic natural isolates in the same species as rhizobia [9,13]. The transfer of symbiosis plasmids in the laboratory can make non-symbiotic isolates symbiotic [14,18] and may convert pathogenic bacteria into symbionts [19]. Root exudates, including plant flavonoids, may enhance the transfer of plasmids or islands between rhizobia [20,21] and transfer may be conditioned by bacterial density sensed by quorum sensing systems [22]. In phylogenetic trees, incongruent phylogenies of symbiosis and core genes are indicative of the natural lateral transfer of symbiosis genes among rhizobia. Consequently, the species name is not indicative of the symbiosis capabilities, although many early names were based on this assumption. The mobility of symbiosis genes means that strains of different species may have the same symbiosis genes and phenotype, while other strains of the same species may have different ones. Discussion and understanding of this situation have been facilitated by giving names to these distinct symbiosis variants, or ‘symbiovars’.

The symbiovar concept can be traced to Jordan’s description of the genus *Rhizobium* in the 1984 edition of Bergey’s Manual [23]. Considering the species *Rhizobium leguminosarum*, which nodulated legumes in the tribe Vicieae, *Rhizobium trifolii*, which nodulated members of the genus *Trifolium*, and *Rhizobium phaseoli*, which nodulated members of the genus *Phaseolus*, Jordan noted that they had no consistent distinguishing characteristics other than host specificity, and there was good evidence that host specificity was conferred by plasmid-borne genes that were transmissible between strains [14,16]. He treated them as a single species, *R. leguminosarum*, with three host-specific biovars. Biovars proved useful for describing host-specificity variants in other rhizobial species, and many were described over the subsequent decades. Eventually, Rogel and collaborators proposed that 'biovar' should be replaced by the more specific term ‘symbiovar’, indicating that it was based on symbiosis specificity for the plant [24]. A symbiovar is characterised by a distinctive set of genes that confer on rhizobia the capability to form nodules in particular plant(s). Single *nod* or *nif* genes would not alone be responsible for the specificity phenotype, but are used as markers representing the set of genes responsible [25].

In pathogenic members of the genus *Agrobacterium*, the term ‘biovar’ was used to refer to genetically distinct species, some of which have been reclassified, such as biovar 2 which is now *Rhizobium rhizogenes* [26,27]. As rhizobia are close relatives of *Agrobacterium,* it was useful to clearly define the term symbiovar (symbiotic variant) in rhizobia as distinct from biovar (biological variant) [24].

The term pathovar is used for pathogenic bacteria to indicate the host affected by a particular species. For pathogenic bacteria, international standards for naming pathovars have been published [28], but there have never been any similar standards for symbiovars. It would be helpful to the rhizobial research community to establish some criteria for the description of symbiovars and to maintain a list of published names. The International Committee for Systematics of Prokaryotes (ICSP) Subcommittee for Rhizobia and Agrobacteria, of which we are members, has tasked us with defining suitable criteria and has agreed to host the list [29].

## Considerations for a stable classification of symbiovars

### The components of a classification system

The requirements for a stable nomenclature of symbiovars should be essentially similar to those for naming bacterial species. It is important to be clear, though, that symbiovars are not part of bacterial taxonomy and are not covered by the International Code of Nomenclature of Prokaryotes [30]. Nevertheless, we still need guidelines for defining symbiovars, naming them and assigning priority. We need to check that the guidelines have been followed, and provide an up-to-date list of accepted symbiovars, with references. Our proposed guidelines are given later in this article, after a discussion of some relevant considerations.

### Recognising symbiovars

Differences in the specificity of interaction with plant hosts were the original rationale for symbiovars, and information on nodulation remains key for their definition. Information on nitrogen fixation and/or other effects on plants is also valuable. Many of the rhizobial genes involved in host specificity have been studied [1,2, 31], but others remain to be defined and could be identified from genome sequences or the results of experimental analyses. It makes sense to include an analysis of symbiosis genes as part of the description of the symbiovar. This is particularly important because some host plants can be nodulated by rhizobia carrying very different symbiosis genes, so a shared host range alone does not guarantee that the genetic basis is similar. If bacteria of different species nodulate the same host, it is important to establish whether they have closely related symbiosis genes that have been passed around by horizontal gene transfer (in which case, it is justified to consider them to be the same symbiovar), or have independently converged on the same host specificity using different genes (in which case it is better to describe them as distinct symbiovars). Distinctive sets of symbiosis genes, and their phylogeny, may also be important for distinguishing symbiovars in the case of rhizobia that have very broad host ranges, as it can be difficult to define differences in host specificity.

Sequences and phylogenies of symbiosis genes (*nod* and *nif*) can be used to identify symbiovars [24]. In bacterial taxonomy, average nucleotide identity (ANI) of the whole genome has proved to be very useful to distinguish distinct species and ANI has become a gold standard [32]. Species boundaries are usually around 95–96 % ANI, but need to be assessed in each case by seeking a minimum in the distribution of ANI values [33]. Similarly, the percentage identity in nucleotide or amino acid sequences of marker symbiosis genes can help to distinguish between distinct symbiovars, but it is not appropriate to set thresholds for individual genes because specificity is determined by the ensemble of *nod* (and, to a lesser extent, *nif*) genes, and not all genes need to be highly diverged. Nevertheless, it is generally true that the symbiosis genes evolve together, and each gene will show less variation within than between symbiovars. Hence, phylogenetic trees of representative genes allow distinct groups to be delineated. Several different individual genes have been used for this purpose, though the majority of authors have included *nodC* and we suggest that a phylogeny of this gene should be included in the description of every new symbiovar. In the genus *Bradyrhizobium*, a limit of 92.5 % identity of *nodC* gene sequences served to distinguish symbiovars *sojae* and *pachyrhizi* [34], and a 92 % threshold was suggested for symbiovars in the genus *Paraburkholderia* [35], similar to the value proposed for symbiovars of *Ensifer fredii* [36]. For a number of other symbiovars in the genus *Bradyrhizobium*, a limit in NifH amino acid identity around 95 % was found to be suitable [25]. However, these genes are not the unique determinants of specificity, as there are other genes involved. Besides the common *nod* and *nif* genes, rhizobial secretion systems, especially type 3 but also type 6, are known to have important roles in host specificity [2,31], so it would be advisable to include them or any other relevant symbiosis gene [37] in the symbiovar description. Since it is now almost as easy to sequence entire bacterial genomes as to sequence individual genes, we think that each symbiovar should be represented by at least one complete genome sequence, so that all the potential host specificity determinants can be explored. It is important that the genome sequence should be of good quality, just as for species descriptions [38], and authors should check that the symbiosis gene sequences, especially of *nodC*, are complete and reliable. For the symbiovars described to date, we have, where possible, selected a representative strain with a sequenced genome, and this should be a requirement for symbiovar description in future.

All symbiovars described so far have a representative strain for which at least the *nodC* sequence is published (and in most cases the genome sequence), with one exception. The representative strain defined by the authors of sv. *medicaginis* is CI4, but they only provided a sequence for *nodA*, not for *nodC* [39]. There is another strain of sv. *medicaginis*, USDA1170, which appears in the original authors' *nodA* tree and has an identical *nodA* sequence to that of CI4. There is a genome sequence available for this strain (GCF_004003925.1), so the *nodC* sequence is available and could be used to represent sv. *medicaginis* in future studies. We have included the genome accession of USDA1170 in Table 1, but have not proposed that this strain should replace CI4 as the representative strain because its host specificity has not been determined experimentally.

**Table 1.** The list of recognised symbiovars

Symbiovar	Bacterial species	Plant hosts	Representative strain	Genome accession number	*nodC* accession number	Authority	Other references
acaciae	*Sinorhizobium terangae, Sinorhizobium sahelense, Sinorhizobium meliloti*	*Acacia* sp., *Acacia tortilis*	USDA 4894	GCF_009601505.1		[57]	[58,60]
acaciellae	*Sinorhizobium chiapanecum, Sinorhizobium mexicanum*	*Acaciella angustissima*	ITTG R7	GCF_017873105.1		[24]	[61,62]
aegeanensis*(was aegeanense)	*Sinorhizobium fredii*	*Vigna unguiculata*	VUKA1	no genome	LT615114	[63]	
africana	*Paraburkholderia sprentiae, Paraburkholderia tuberum, Paraburkholderia dilworthii, Paraburkholderia rynchosiae, Paraburkholderia stridomiana, Paraburkholderia kirstenboschensis, Paraburkholderia dipogonis, Paraburkholderia steynii*	*Aspalathus carnosa, Lebeckia ambigua, Dipogon lignosus, Virgilia oroboides, Rhynchosia ferulifolia,* rhizosphere of *Hypocalyptus sophoroides*	WSM5005	GCF_000473465.1		[35]	
americensis*^,†^(was americaense)	*Bradyrhizobium yuanmingense*	*Cajanus cajan*	ALSPC3020	GCF_029167055.1		[64]	
anthyllidis	*Mesorhizobium delmonti, M. prunaderense, M. metallidurans, M.ventifaucium, M. escarrei*	*Anthyllis vulneraria*	STM 2683	GCF_000350085.1		[65]	[66]
arachidis	*Bradyrhizobium zhengyangense, Bradyrhizobium guangdongense, Bradyrhizobium guangxiense, Bradyrhizobium guangzhouense*	*Arachis hypogaea*	CCBAU 051107	GCF_015291705.1		[67]	
astragali	*Mesorhizobium qingshengii, Mesorhizobium huakuii, Mesorhizobium jarvisii*	*Astragalus sinicus*	7653R	GCA_000709395.2		[68]	
atlantica	*Paraburkhloderia atlantica*	*Mimosa pigra, Mimosa velloziana, Mimosa occidentalis, Mimosa pudica, Calliandra parvifolia*	CNPSo 3155	GCF_009362785.1		[35]	
biserrulae	*Mesorhizobium ciceri, Mesorhizobium australicum, Mesorhizobium opportunistum*	*Biserrula pelecinus*	WSM1271	GCF_000185905.1		[69]	
cajani	*Bradyhizobium cajani* and nine other species	*Cajanus cajan, Arachis hypogaea, Chamaecrista fasciculata, Erythrina brucei, Glycine clandestina, Vigna unguiculata, Inga* sp.	AMBPC1010	GCF_009759665.1		[70]	
canariensis*(was canariense)	*Mesorhizobium neociceri*	*Cicer canariense*	CCANP35	GCF_013520985.1		[56]	
caraganae	*Mesorhizobium caraganae*	*Cicer canariense*	LMG 24397	GCF_016836705.1		[56]	
caribensis*(was caribense)	*Bradyrhizobium yuanmingense*	*Cajanus cajan*	ALPC2061	GCF_029167075.1		[64]	
cenepequi	*Bradyrhizobium cenepequi*	*Vigna unguiculata*	CNPSo 4026	GCF_020329485.1		[70]	
centrosemae	*Bradyhizobium centrosematis*	*Centrosema*	A9	no genome	KC247134	[40]	
ciceri	*Mesorhizobium ciceri, Mesorhizobium wenxiniae, Mesorhizobium amorphae*	*Cicer arietinum*	LMG 14989	GCF_029269405.1		[71]	[68]
cyanophyllae	*Bradyrhizobium* spp.	*Acacia saligna*	1AS2L	no genome	OM429516	[72]	
fredii	*Sinorhizobium fredii*	*Glycine max*	CCBAU 25509	GCF_003177055.1		[45]	
gallica*(was gallicum)	*Rhizobium azibense, Rhizobium gallicum*	*Phaseolus vulgaris, Mimosa, Sesbania* and others	R602sp	GCF_000816845.1		[73]	[74]
genistearum	*Bradyhizobium canariense, Bradyhizobium hipponense, Bradyhizobium retamae, Bradyhizobium rifense*, *Bradyhizobium lupini, Bradyhizobium cytisi*	*Genista, Leucaena leucocephala, Chamaecytisus, Lupinus, Cytisus villosus*	BTA-1	GCF_019402665.1		[75]	[76,78]
giardinii	*Pararhizobium giardinii*	*Phaseolus vulgaris*	H152	GCF_000379605.1		[73]	
glycinearum	*Bradyhizobium japonicum, Bradyhizobium daqingense, Bradyhizobium nanningense, Bradyhizobium barrani, Bradyhizobium ottawaense, Bradyhizobium huanghuaihaiense, Bradyhizobium diazoefficiens*	*Glycine soja, Arachis hypogaea*	USDA 6	GCF_000284375.1		[79]	[34,70, 80, 81]
glycinis	*Bradyhizobium glycinis*	*Glycine tabacina*	CNPSo 4016	GCF_016031655.1		[70]	
glycyphyllae	*Mesorhizobium* ssp.	*Astragalus glycyphyllos*	AG1	no genome	KR004129	[82]	
hedysari	*Mesorhizobium camelthorni*	*Lotus creticus, Sulla spinosissima*	LCD11	no genome	MN418121	[53]	[54]
ingae	*Bradyrhrhizobium ingae, Bradyrhizobium* spp.	*Inga laurina, Inga cayennensis, Inga ingoides*	CCGUVB23	GCF_024199885.1		[25]	
lancerottensis*(was lancerottense)	*Sinorhizobium meliloti*	*Lotus creticus, Lotus lancerottensis*	Llan-2	no genome	FM178855	[83]	[84,85]
loti	*Mesorhizobium loti, Mesorhizobium japonicum, Mesorhizobium helmanticense, Mesorhizobium tamadayense, Mesorhizobium erdmanii, Mesorhizobium olivaresii, Mesorhizobium sanjuani*	*Lotus berthelotii, Lotus corniculatus, Lotus japonicus, Lotus tenuis, Lotus lancerottensis*	MAFF 303099	GCF_000009625.1		[86]	[41,55, 87,90]
lupini	*Bradyhizobium valentinum, Bradyhizobium retamae*	*Lupinus micranthus*	LmiH4	no genome	KX272805	[47]	
lysilomae	*Bradyrhizobium* spp.	*Lysiloma* sp.	CCGB20	GCF_024199855.1		[25]	
lysilomefficiens†(was lysilomaefficiens)	*Bradyrhizobium* sp.	*Lysiloma* sp.	CCGB12	GCF_024199845.1		[25]	
maamorensis†(was maamori)	*Mesorhizobium* sp.	*Ononis repens*	ORM6	no genome	OM649869	[42]	
magrebensis‡(was mediterranense)	*Phyllobacterium sophorae, Microvirga tunisiensis*	*Lupinus micranthus*	LmiT21	no genome	KX272804.1	[47]	[48,49]
medicaginis	*Sinorhizobium meliloti*	*Medicago laciniata*	CI4	GCF_004003925.1§	*nodA*: AJ441287	[39]	
mediterranensis*(was mediterranense)	*Sinorhizobium americanum*	*Phaseolus vulgaris*	4H41	GCF_000375585.1		[45]	[46,91]
meliloti	*Sinorhizobium meliloti, Sinorhizobium medicae*	*Medicago sativa, Medicago truncatula, Trigonella phoenum-graecum, Lotus creticus, Lotus pusillus*	1021	GCF_000006965.1		[39]	[53,92]
mimosae	*Rhizobium etli, Rhizobium phaseoli*	*Mimosa*	Mim1	GCF_000442435.1		[50]	[24,51]
mimosoidearum‡(was mimosae)	*Paraburkholderia guartelaensis, Paraburkholderia nodosa, Paraburkholderia mimosarum*	*Mimosa pudica, Mimosa pigra, Mimosa scabrella, Mimosa gymnas*	CNPSo 3008	GCF_004353905.1		[35]	
officinalis	*Neorhizobium galegae*	*Galega officinalis*	HAMBI 1141	GCF_000731295.1		[43]	
orientalis	*Neorhizobium galegae*	*Galega orientalis*	HAMBI 540	GCF_000731315.1		[43]	
oxytropidis†(was oxytropis)	*Mesorhizobium* sp., *Mesorhizobium loti, Mesorhizobium huakuii*	*Oxytropis nigrescens*	AR02	GCF_024746835.1		[41]	
pachyrhizi	*Bradyrhizobium pachyrhizi*	*Acacia auriculiformis, Parasponia*	PAC 48	GCF_001189245.1		[34]	
phaseolearum†(was phaseolarum)	*Bradyrhizobium americanum*	*Centrosema macrocarpum*	CMVU44	no genome	KC247130.1	[40]	
phaseoli	*Rhizobium anhuiense, Rhizobium acidisoli, Rhizobium chutanense, Rhizobium ecuadorense, Rhizobium etli, Rhizobium esperanzae, Rhizobium hidalgonense, Rhizobium leguminosarum* complex*, Rhizobium lusitanum, Rhizobium phaseoli, Rhizobium vallis, Rhizobium sophoriradicis*	*Phaseolus vulgaris, Sophora flavescens*	CFN 42	GCF_000092045.1		[23]	[73, 93,106]
piptadeniae	*Paraburkholderia ribeironis*	*Piptadenia gonoacantha*	STM7296	GCF_900019265.1		[35]	
polyphytorum‡(was orientale)	*Rhizobium gallicum*	*Medicago ruthenica, Coronilla varia, Hedysarum* sp.*,* and others	USDA 1844	GCF_000419765.1		[44]	
psoraleae	*Mesorhizobium chacoense, Mesorhizobium jarvisii*	*Bituminaria bituminosa*	ICMP12638	no genome	KM018083	[107]	
retamae	*Bradyhizobium lablabi, Bradyhizobium retamae, Bradyhizobium archetypum, Bradyhizobium australiense, Bradyhizobium hereditatis, Bradyhizobium murdochi, Bradyhizobium paxllaeri, Bradyhizobium namibiense, Bradyhizobium icense, Bradyhizobium algeriense, Bradyhizobium valentinum*	*Lablab, Retama monosperma, Retama sphaerocarpa, Muelleranthus, Indigofera, Rhynchosia, Phaseolus lunatum, Lupinus mariae-josephae*	Ro19	GCF_001440415.1		[108]	[70]
rigiduloides	*Sinorhizobium meliloti*	*Medicago rigiduloides*	M270	GCF_002197085.1		[109]	[110]
salignae	*Rhizobium acaciae*	*Acacia saligna*	1AS11	GCF_025941625.1		[72]	[111]
septentrionalis*(was septentrionale)	*Bradyhizobium septentrionale, Bradyhizobium quebecense*	*Glycine max, Amphicarpaea bracteata, Desmodium glutinosum*	66S1MB	GCF_013373795.3		[112]	
sesbaniae	*Mesorhizobium hawassense, Sinorhizobium saheli, Sinorhizobium terangae*	*Sesbania sesban, Sesbania cannabina*	LMG 7837	GCF_001651875.1		[57]	[41,113]
sierranevadensis*(was sierranevadense)	*Bradyrhizobium* sp.	*Genista versicolor*	GV137	no genome	KF483556.1	[114]	
sojae	*Bradyhizobium elkanii, Bradyhizobium ivorense, Bradyhizobium ferriligni*	*Glycine max, Cajanus cajan, Erythrophleum fordii*	USDA 76	GCF_023278185.1		[34]	
trifolii	*Rhizobium anhuiense, Rhizobium hidalgonense, Rhizobium acidisoli, Rhizobium bangladeshense, Rhizobium aethiopicum, Rhizobium pisi, Rhizobium leguminosarum* complex	*Trifolium* spp., *Trifolium alexandrinum, Phaseolus vulgaris, Cicer canariense*	TA1	GCF_021052425.1		[23]	[104,115,117]
tropicalis	*Paraburkhoderia franconis, Paraburkhoderia caribense, Paraburkhoderia phymatum, Paraburkhoderia diazotrophica, Paraburkhoderia piptadeniae*	*Mimosa flocculosa, Mimosa candollei, Mimosa caesalpiniifolia, Mimosa diplotricha, Mimosa pudica, Machaerium lunatum, Piptadenia gonoacantha*	CNPSo 3157	GCF_009362735.1		[35]	
tropici	*Rhizobium tropici, Rhizobium freirei, Rhizobium leucaenae, Rhizobium lusitanum, Rhizobium* sp., *Bradyrhizobium pachyrhizi*	*Leucaena leucocephala, Phaseolus vulgaris, Macroptilium atropurpureum* and others	CIAT 899	GCF_000330885.1		[5]	[100,118,121]
tropiciagri‡(was tropici)	*Bradyrhizobium viridifuturi, Bradyrhizobium embrapense, Bradyrhizobium* spp.	*Centrosema*	SEMIA 690	GCF_001238275.1		[34]	[40]
vachelliae	*Sinorhizobium fredii, Sinorhizobium arboris, Sinorhizobium aridi*	*Vachellia gummifera, Prosopis chilensis, Acacia cyanophylla, Leucaena leucocephala*	LMG 14919	GCF_000427465.1		[36]	
viciae	*Rhizobium anhuiense, Rhizobium leguminosarum* complex*, Rhizobium pisi, Rhizobium multihospitium, Rhizobium acidisoli, Rhizobium binae, Rhizobium lentis, Rhizobium bangladeshense*	*Pisum sativum, Lens culinaris, Vavilovia formosa, Vicia* spp.*, Lathyrus* spp.*, Phaseolus vulgaris*	3841	GCF_000009265.1		[23]	[104,117, 122,124]
vignae	*Bradyrhizobium* sp. (group II), *B. australafricanum*	*Vigna unguiculata, Glycine* sp.	VUPME10	no genome	HG940520.1	[125]	

1* nName in feminine form.

2† sSuggested corrected name.

3‡ pProposed replacement for duplicate name.

§Genome sequence of strain USDA1170 (see text).

### Naming symbiovars

Symbiovar names proposed so far are in a Latin style similar to species epithets. Symbiovar is not a taxonomic category at all, but a description of a phenotype that is conferred by a set of genes that is potentially mobile between species and even between genera. Thus, symbiovar names can be considered to describe the word ‘symbiovar’. For example, ‘symbiovar lancerottensis’ is the symbiovar from Lanzarote, whichever rhizobial genus it occurs in. Since the word ‘symbiovar’ is derived from the Latin ‘varietas’, which is feminine, we propose that all symbiovar names should reflect the feminine gender regardless of the bacterial genus they are found in. In the past, symbiovar names were proposed that agreed with the gender of the genus, which was most often neuter. Rather than make an immediate ‘correction’ of these names, we list them in Table 1 both in the familiar neuter form (e.g. ‘-ense’) and in the feminine form (e.g. ‘-ensis’) that we think is more appropriate for the future. We consider the two forms to be synonymous.

There are a few symbiovar names that, if they had been proposed as species epithets, would have been corrected by the nomenclature editors because they are not well formed according to the rules for Latin names. Thus, *lysilomaefficiens* [25] would be better as *lysilomefficiens* (based on the stem *Lysilom-* of the host plant name), *phaseolarum* [40] would be *phaseolearum* (of the tribe Phaseoleae), *oxytropis* [41] would be *oxytropidis* (of *Oxytropis*), *maamori* [42] would be *maamorensis* (from the place Maamora). While authors may, in future, prefer to use the corrected form, we consider either version to be acceptable.

Symbiovar names should be unique, even those that are found in different genera, because one of their key features is that they can be shared between species and even between genera. We need to resolve several cases of duplicate names that are already published. The name *orientalis/orientale* (differing only in gender) has been used twice, first for strains of *Neorhizobium galegae* that are symbionts of *Galega orientalis *[43] and later for* Rhizobium mongolense* and *Rhizobium yanglingense* symbionts of various wild legume species [44]. To resolve this, we propose to change the name of the later symbiovar to sv. *polyphytorum*, since it has been isolated from many different host plant species. The name sv. *tropici* has also been used twice, first for broad host-range strains of *Rhizobium tropici*, *Rhizobium leucaenae* and *Rhizobium lusitanum* [5] and later for *Bradyrhizobium* symbionts of *Centrosema* species and other legumes [40]. We propose that this second symbiovar be renamed sv. *tropiciagri*, since *Bradyrhizobium tropiciagri* is one of the species in which it is found. The name sv. *mediterranense* was defined for *Sinorhizobium* symbionts of *Phaseolus vulgaris* [45,46], but later the same name was used to describe a strain of *Phyllobacterium sophorae* that nodulated *Lupinus micranthus* in Tunisia [47], and this last symbiovar was also found later in *Microvirga tunisiensis* strains from both Tunisia and Morocco that nodulate various *Lupinus* species [48,49]. We propose that this later symbiovar be renamed *magrebensis*, reflecting its isolation from two countries in the Maghreb region (northwest Africa). The name sv. *mimosae* was first used to describe strains of *R. etli* that nodulate members of the genus *Mimosa* [50,51], but the same name was later applied to strains of *Paraburkholderia* that also nodulated *Mimosa* species but using very different symbiosis genes, so they are clearly a different symbiovar [52]. We propose that the symbiovar found in *Paraburkholderia* be renamed sv. *mimosoidearum* (of the traditional subfamily Mimosoideae).

We also need to resolve cases where the same symbiovar has been defined with different names, as is the case of symbiovar *hedysari/aridi*, defined in *Mesorhizobium camelthorni *first as sv. *hedysari* [53] and some months later as sv. *aridi* [54]. In this case, considering priority for the date of definition, we should maintain *hedysari* and consider *aridi* as a later synonym. Hence, sv. *aridi* is not listed in Table 1.

The proposal of a new symbiovar name needs to be accompanied by a discussion of the evidence that it is novel and distinct, including information on host specificity and on gene sequences. The names sv. *albiziae*, sv. *robiniae*, sv. *sophorae* and sv. *astragalus/caraganae* have been used to label clades in a *nodC* phylogeny [55], but the authors did not describe them in the text, so we consider that they did not define these potential symbiovars and we have not included the names in Table 1. Several later publications have repeated the names [41,42, 56], but still with no formal definition.

When Jordan [23] first described biovars in *R. leguminosarum*, he used italics for the names, but when Rogel *et al*. [24] introduced the more specific term symbiovar they did not use italics. Both styles have been followed extensively in the literature, and we consider that it is equally acceptable to write symbiovar names with italics or without.

## Proposed guidelines for the description and naming of symbiovars

### Description

1a. Plant specificity is the essential basis to define a symbiovar. Symbiovars should be described in relation to the symbiosis phenotypes, not only referring to nodulation but also to effectiveness by considering nitrogen fixation.

1b. A symbiovar must have a representative strain, and the genome sequence of that strain must be publicly available and of sufficient quality [38]. It is recommended (but not obligatory) that the representative strain be available in an international culture collection.

1c. It must be demonstrated that the symbiosis genes are recognisably distinct from those of all previously described symbiovars in sequence or in the presence/absence of specific genes.

### Naming

2a. The name of a new symbiovar must be distinct from all previously recognised symbiovar names in all genera.

2b. Symbiovar names that are adjectival always take the feminine gender.

2c. In other respects, the names of symbiovars follow the same rules as those of species epithets.

2d. A proposal for a new symbiovar must be published in the body (not supplementary files) of an article in a peer-reviewed journal, and must include all the information required by these guidelines.

### Recognition

3a. The ICSP Subcommittee for Rhizobia and Agrobacteria undertakes to maintain a list of recognised symbiovars and to appoint persons to manage this.

3b. After publication, authors should submit the publication to the Secretary of the ICSP Subcommittee for Rhizobia and Agrobacteria.

3c. New symbiovars that conform to the guidelines and have priority will be added to the online list of recognised symbiovars on the Subcommittee website (currently https://sites.google.com/view/taxonomyagrorhizo/home).

3d. The priority of names is established by the date of inclusion in the list.

## List of recognised symbiovars

A list of all symbiovars (in alphabetical order) proposed to date is given in Table 1. For each symbiovar, references are given for the name and description, for the rhizobial species in which it has been found naturally, and for the range of legume hosts that are nodulated. In most cases, the original authors did not specify a representative strain; whenever possible, we have selected one with an available genome sequence.

## Conclusion

In view of the large number of symbiovars described so far, and the likelihood that the number will increase in the future when more legumes are thoroughly studied, we felt the need to consider some appropriate standards and to clarify the status of the symbiovars already described. We have proposed guidelines for the description and naming of symbiovars and offered to set up and maintain a public list as a service to the rhizobial research community. Our aim is to make it easier to understand and use the symbiovar concept. As with any new venture, we may find we need to make adjustments in the light of experience, and we welcome feedback from users.

## Abbreviations

ANI: average nucleotide identity
HGT: horizontal gene transfer
ICSP: International Committee for Systematics of Prokaryotes
sv: symbiovar
